# Left Ventricular Assist Device Implantation and Concomitant Dor Procedure: a Single Center Experience

**DOI:** 10.21470/1678-9741-2019-0349

**Published:** 2020

**Authors:** Andreas Schaefer, Yvonne Schneeberger, Liesa Castro, Bjoern Sill, Yousuf Alassar, Meike Rybczynski, Markus J Barten, Hanno Grahn, Hermann Reichenspurner, Sebastian A Philipp, Alexander M Bernhardt

**Affiliations:** 1 Department of Cardiovascular Surgery, University Heart and Vascular Center Hamburg, Hamburg, Germany.; 2 Department of General and Interventional Cardiology, University Heart and Vascular Center Hamburg, Hamburg, Germany.; 3 Department of Cardiology and Intensive Care Medicine, Elbe Clinic, Stade, Germany.

**Keywords:** Heart Transplantation, Heart Failure, Thrombosis, Thromboembolism, Aneurysm, Cardiomyopathies

## Abstract

**Objective:**

Left ventricular assist device (LVAD) implantation with concomitant Dor plasty is only reported anecdotally. We herein aimed to describe our experience with LVAD and concomitant Dor procedures and describe long-term outcomes of this special subset of heart failure patients.

**Methods:**

Between January/2010 and December/2018, 144 patients received LVAD therapy at our institution. Of those, five patients (80% male, 60.4±7.2 years) presented with an apical aneurysm and received concomitant Dor plasty. Apical aneurysms presented diameter between 75 and 98 mm, with one impending rupture.

**Results:**

Procedural success was achieved in all patients. No unplanned right ventricular assist device implantation occurred. Furthermore, no acute 30-day mortality was seen. In follow-up, one patient was lost due to intentional disconnection of the driveline. One patient underwent heart transplantation on postoperative day 630. The remaining three patients are still on device with sufficient flow; pump thromboses were successfully managed by lysis therapy in one patient.

**Conclusion:**

LVAD implantation with concomitant Dor procedure is feasible, safe, and occasionally performed in patients with ischemic cardiomyopathy. Major advantages are prevention of thromboembolism and facilitation of LVAD placement by improving pump stability and warranting midventricular, coaxial alignment of the inflow cannula. In long-term follow-up, no adverse event associated with Dor plasty was observed.

**Table:** 

Abbreviations, acronyms & symbols				
AKI	= Acute kidney injury		ICMP	= Ischemic cardiomyopathy
AV	= Aortic valve		INTERMACS	= Interagency Registry for Mechanically Assisted Circulatory Support
BMI	= Body mass index		LDH	= Lactic acid dehydrogenase
BTR	= Bridge to recovery		LV	= Left ventricle
BTT	= Bridge to transplantation		LVAD	= Left ventricular assist device
CABG	= Coronary artery bypass grafting		LVEDD	= Left ventricular end-diastolic diameter
CF	= Continuous-flow		LVEF	= Left ventricular ejection fraction
COPD	= Chronic obstructive pulmonary disease		MCS	= Mechanical circulatory support
CPB	= Cardiopulmonary bypass		MI	= Mitral insufficiency
CRP	= C-reactive protein		MV	= Mitral valve
CVVHD	= Chronic venovenous hemofiltration		PCI	= Percutaneous coronary intervention
DT	= Destination therapy		RVAD	= Right ventricular assist device
ECMO	= Extracorporeal membrane oxygenation		RVP	= Right ventricular pressure
EVPP	= Endoventricular patch plasty		s/p	= Status post
GOLD	= Global Initiative for Chronic Obstructive Lung Disease		STEMI	= ST-elevation myocardial infarction
GOT	= Glutamate-oxaloacetate transaminase		TAPSE	= Tricuspid annular plane systolic excursion
GPT	= Glutamate-pyruvate transaminase		Thr	= Thrombus
HF	= Heart failure		TI	= Tricuspid insufficiency
HTx	= Heart transplantation		VT	= Ventricular fibrillation
IABP	= Intra-aortic balloon pump			
ICD	= Implantable cardioverter defibrillator			

## INTRODUCTION

Due to limited availability of donor organs for heart transplantation, implantation of intracorporal miniaturized left ventricular assist devices (LVAD) became clinical daily routine for treatment of end-stage heart failure (HF), with the number of procedures increasing annually^[1,2]^. HF remains the most common reason for hospital admission in the United States and Western Europe, and therefore, it can be anticipated that the number of LVAD implantations will further increase. Currently, 100% of patients in need for destination therapy, registered in the Interagency Registry for Mechanically Assisted Circulatory Support (INTERMACS), receive continuous-flow (CF) pumps^[3,4]^. These miniaturized CF pumps improve the outcome of patients with end-stage HF in terms of symptoms, hospitalization, and premature death while awaiting transplantation^[5]^. Patients selected for LVAD implantation usually suffer from significant comorbidities which may (*e.g*., renal failure and lung disease) or may not be associated with the underlying cardiac disease^[6]^. Especially concomitant cardiac conditions, like valve stenosis/regurgitation, coronary heart disease, or right ventricular failure, require additional procedures during LVAD implantation. Here, outcomes were reported for concomitant valve repair/replacement (aortic, mitral, tricuspid), patent foramen ovale closure, or implantation of a temporary right ventricular assist device (RVAD)^[7-9]^. Another precondition which may be complicating to LVAD surgery is a left ventricular aneurysm in patients with ischemic cardiomyopathy (ICMP) and status post (s/p) transmural myocardial infarction. Here, LVAD insertion is compounded by a thin apical myocardium, and left ventricular thrombus formation is often present^[10]^. To facilitate CF pump insertion and placement and to prevent thromboembolism in such cases, concomitant endoventricular patch plasty (EVPP) (Dor procedure^[11]^) should be taken into consideration. However, these combined procedures are only described anecdotally with documentation of acute and perioperative outcomes^[12]^. We herein describe our experience with combined LVAD and Dor procedures with an emphasis on long-term follow-up.

## METHODS

### Patients

Between January/2010 and December/2018, 144 patients received LVAD therapy for end-stage HF at our institution. Of those, five patients (5/144, 3.5%) presented with apical aneurysms and received concomitant Dor plasty during LVAD implantation. All five patients presented with ICMP, s/p ST-elevation myocardial infarction, and severely reduced left ventricular ejection fraction. Four patients had a history of multiple percutaneous coronary interventions and one patient underwent multiple coronary artery bypass grafting procedures.

### Ventricular Assist Device Implantation and Concomitant Endoventricular Patch Plasty

**Fig. 1:**
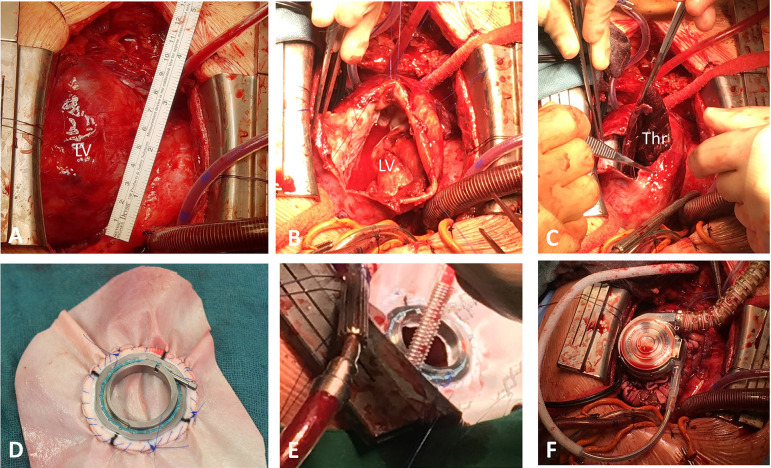
Intraoperative pictures of left ventricular assist device (LVAD) implantation with concomitant Dor plasty. Intraoperative course of patient n. 5: large left ventricular apex aneurysm after median sternotomy (A), opening of the apex (B) and removal of a large thrombus (Thr) (C), adaptation of the LVAD sewing ring to a patch (D), apex reconstruction with the patch (E), and implantation of the LVAD device (F). LV=left ventricle

In all patients, a HeartWare HVAD (Medtronic, Minneapolis, MN, USA) device was implanted through median sternotomy with support of cardiopulmonary bypass (CPB) to unload the left ventricle. After luxation of the heart, the left ventricular apex was opened. Subsequently, thrombotic material was removed, and the edge of vital myocardium was identified. Here, a reinforcing running 2-0 Prolene (Ethicon Inc., Somerville, NJ, USA) suture buttressed with Teflon felt was performed. Simultaneously, the HVAD ring was sewed in a Vascutek (Terumo Co., Shibuya, Tokyo, Japan) or pericardial patch utilizing a running 3-0 Prolene suture. Then, the patch was attached to the neoapex with interrupted felt-pleged 3-0 Prolene sutures and the patch was furthermore secured by a subsequent performed running 2-0 Prolene suture comprising transmural stitches of vital myocardium. Further procedural steps followed institutional routines including HVAD pump insertion and alignment, tunneling of the driveline, and attaching of the outflow graft to the ascending aorta. Crucial steps of the described procedure are depicted in Figure 1.

### Follow-Up

Baseline, intraprocedural, and follow-up data were collected and entered into a dedicated standardized database. Clinical endpoints were death and heart transplantation. Mean follow-up time was 547.6±473.1 days. Data are presented as absolute numbers and percentages for categorical variables and mean values and standard deviation for continuous variables, unless stated otherwise.

## RESULTS

### Baseline Demographics

Overall, five patients (80% male, 60.4±7.2 years) received concomitant EVPP during LVAD implantation for apical aneurysm and end-stage HF at our institution. Of those, three patients (60%) fulfilled clinical requirements for INTERMACS level 1 or 2, with one patient in need for preoperative temporary mechanical circulatory support (extracorporeal membrane oxygenation therapy). Two patients presented with INTERMACS levels 3 and 4. In three patients, LVAD implantation strategy was bridge to transplantation, and in two patients destination therapy.

Detailed baseline patient demographics are shown in Table 1.

**Table 1:** Baseline characteristics of patients who underwent LVAD implantation with concomitant Dor plasty.

	Patient Nº.					
	1	2	3	4	5	Σ
Age, years	66	49	61	67	59	60.4±7.2
Gender, male/female	Female	Male	Male	Male	Male	4/5 male
BMI, kg/m^2^	26.7	21.6	28.9	22.3	24.1	24.7±3.1
INTERMACS profile	2	1	3	2	4	/
Strategy for LVAD implantation, **✓**/**✘**						/
BTT	**✘**	**✓**	**✓**	**✘**	**✓**	/
DT	**✓**	**✘**	**✘**	**✓**	**✘**	/
BTR	**✘**	**✘**	**✘**	**✘**	**✘**	/
Preoperative mechanical ventilation, **✓**/**✘**	**✘**	**✓**	**✘**	**✘**	**✘**	/
Preoperative MCS (ECMO, IABP, Impella)	**✘**	ECMO	**✘**	**✘**	**✘**	/
Further diagnosis	s/p STEMI, s/p PCI, COPD, GOLD IV, s/p breast cancer	s/p STEMI, s/p PCI, s/p stroke, diabetes mellitus	s/p STEMI, s/p CABG, re-CABG, re-re-CABG, s/p PCI, s/p stroke	s/p STEMI, s/p PCI, s/p dilative tracheostomy, s/p alcohol abuse	s/p STEMI, s/p PCI	/

BMI=body mass index; BTR=bridge to recovery; BTT=bridge to transplantation; CABG=coronary artery bypass grafting; COPD=chronic obstructive pulmonary disease; DT=destination therapy; ECMO=extracorporeal membrane oxygenation; GOLD=Global Initiative for Chronic Obstructive Lung Disease; IABP=intra-aortic balloon pump; INTERMACS=Interagency Registry for Mechanically Assisted Circulatory Support; LVAD=left ventricular assist device; MCS=mechanical circulatory support; PCI=percutaneous coronary intervention; s/p=status=post; STEMI=ST-elevation myocardial infarction

### Preoperative Laboratory and Echocardiographic Findings

Preoperatively, patients presented with moderate signs of end-organ damage in advanced HF with a mean creatinine value of 1.3±0.6 mg/dl and a glutamic oxaloacetic transaminase of 113.6±191.2 U/l.

Echocardiography revealed a preoperative left ventricular ejection fraction of 16.2±5.1% and no precursors of impaired right ventricular function with a tricuspid annular plane systolic excursion of 17.0±4.5 mm, a right ventricular pressure of 45.2±6.4 mmHg, and a tricuspid insufficiency ≥ moderate in only one patient (1/5, 20%).

Apical aneurysms presented with a diameter between 75 and 98 mm, with left ventricular end-diastolic diameters between 44 and 70 mm. One patient with an apical aneurysm of 98 mm presented an extremely thin myocardial wall of the left ventricular apex in preoperative echocardiography, and LVAD implantation with concomitant EVPP was performed as an emergency procedure due to risk of rupture.

**Fig. 2:**
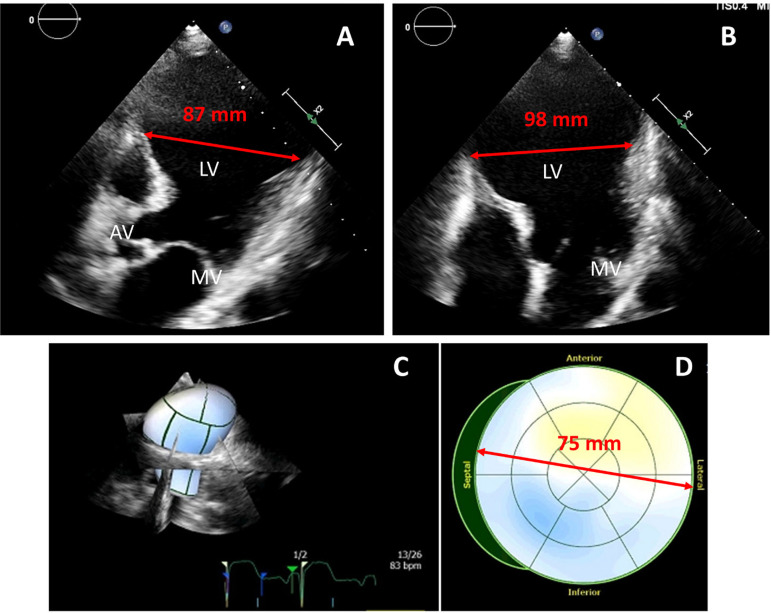
Preoperative transthoracic echocardiography with depiction of large left ventricular apex aneurysms in patients prior to left ventricular assist device implantation with concomitant Dor plasty. Five-chamber view of patient n. 1 with a left ventricular apex aneurysm of 87 mm (A), fourchamber view of patient n. 5 with a left ventricular apex aneurysm of 98 mm and impending rupture (B), and 3D left ventricular apex reconstruction of patient n. 4 with a septolateral diameter of 75 mm (C, D). AV=aortic valve; LV=left ventricle; MV=mitral valve

Representative preoperative echocardiographic images of apical aneurysms are shown in Figure 2.

Detailed preoperative laboratory and echocardiographic findings are summarized in Table 2.

**Table 2:** Periprocedural laboratory, echocardiographic, and intraoperative characteristics of patients who underwent LVAD implantation with concomitant Dor plasty.

	Patient No.					
	1	2	3	4	5	Σ
Laboratory						
Creatinine, mg/dl	0.9	2.0	0.9	1.8	0.8	1.3±0.6
Hemoglobin, g/dl	9.2	9.1	10.9	13.9	10.6	10.7±1.9
GOT, U/l	13	44	29	455	27	113.6±191.2
GPT, U/l	20	34	47	80	63	48.8±23.6
Lactate, mmol/l	1.5	2.4	2.1	2.8	1.1	1.9±0.7
CRP, mg/l	5	82	9	47	24	33.4±31.8
LDH, U/l	165	235	226	123	319	213.6±74.7
Echocardiography						
LVEF, %	15	12	25	14	15	16.2±5.1
LVEDD, mm	66	70	64	68	44	62.4±10.5
Apical aneurysm diameter, mm	87	98	77	75	98, impending rupture	84.3±10.6
MI ≥ grade 2, **✓**/**✘**	**✘**	**✓**	**✘**	**✘**	**✘**	/
TI ≥ grade 2, **✓**/**✘**	**✘**	**✘**	**✘**	**✘**	**✓**	/
RVP, mmHg	47	55	44	42	38	45.2±6.4
TAPSE, mm	23	12	16	20	14	17.0±4.5
Procedure time, min	305	335	380	260	435	343.0±67.5
CPB time, min	180	195	302	139	224	208.0±60.8
Cross-clamp time, min	0	120	0	0	0	/
Need for temporary RVAD, **✓**/**✘**	**✘**	**✘**	**✘**		**✘**	0/5**✓**

CPB=cardiopulmonary bypass; CRP=C-reactive protein; GOT=glutamate-oxaloacetate transaminase; GPT=glutamate-pyruvate transaminase; LDH=Lactic acid dehydrogenase; LVAD=left ventricular assist device; LVEDD=left ventricular end-diastolic diameter; LVEF=left ventricular ejection fraction; MI=mitral insufficiency; RVAD=right ventricular assist device; RVP=right ventricular pressure; TAPSE=tricuspid annular plane systolic excursion; TI=tricuspid insufficiency

### Periprocedural Data

Mean operation time was 343.0±67.5 min with use of CPB in all cases, with a mean time of 208.0±60.8 min. Cross-clamping of the ascending aorta was performed in one patient. In no case concomitant valve procedures were performed. Also, no planned or unplanned implantation of temporary RVAD was necessary. In all patients, intraoperative echocardiography presented midventricular and coaxial alignment of the LVAD inflow cannula at the end of the procedure.

### Clinical Outcome Data

LVAD implantation success and 30-day survival were 100% (5/5) with no pump thrombosis or major bleedings (including rethoracotomies) in acute follow-up. In two patients, dilative tracheostomy was performed due to respiratory failure with ventilation times of 232 and 790 hours. Both patients were weaned successfully from mechanical ventilation. Chronic venovenous hemofiltration with a duration of 30 days was necessary in one patient with a preoperative creatinine of 2.0 mg/dl.

In long-term follow-up, one patient was lost on postoperative day 98 due to intentional disconnection of the driveline by the patient. One patient underwent orthotopic heart transplantation on postoperative day 630. Three patients are still on device, with gastrointestinal bleedings in two patients. Moreover, one acute kidney injury occurred during follow-up and one patient presented three consecutive pump thromboses, which were successfully managed by lysis therapy. Quarterly performed echocardiography presented stable, coaxial, and midventricular position of the inflow cannula in all patients. Also, flow and revolutions per minute measurements showed steady values.

Detailed outcome data are summarized in Table 3.

**Table 3:** Acute outcomes and follow-up of patients who underwent LVAD implantation with concomitant Dor plasty.

	Patient No.					
	1	2	3	4	5	Σ
LVAD implantation success, **✓**/**✘**	**✓**	**✓**	**✓**	**✓**	**✓**	5/5**✓**
30-day follow-up, LVAD, **✓**/**✘**						
Pump thrombosis	**✘**	**✘**	**✘**	**✘**	**✘**	0/5**✓**
Major bleeding	**✘**	**✘**	**✘**	**✘**	**✘**	0/5**✓**
Dilative tracheostomy	**✘**	**✘**	**✓**	**✓**	**✘**	2/5**✓**
Ventilation time, hours	9	18	232	790	2	210.2±338.2
CVVHD	**✘**	**✓**	**✘**	**✘**	**✘**	1/5**✓**
Duration of CVVHD, days	/	30	/	/	/	/
30-day survival, **✓**/**✘**	**✓**	**✓**	**✓**	**✓**	**✓**	5/5**✓**
Follow-up time, days	540	630	1290	98	180	547.6±473.1
Outcome at follow-up	Alive, on LVAD	Alive, s/p HTx	Alive, on LVAD	Deceased	Alive, on LVAD	/
Complications during follow-up	AKI, gastrointestinal bleeding	Epistaxis, VF, ICD implantation	Gastrointestinal bleeding, 3' pump thrombosis	Intentional disconnection of driveline	/	/

AKI=acute kidney injury; CVVHD=chronic venovenous hemofiltration; HTx=heart transplantation; ICD=implantable cardioverter defibrillator; LVAD=left ventricular assist device; VT=ventricular fibrillation

## DISCUSSION

The main findings of the herein conducted study are: (I) LVAD implantation with concomitant Dor procedure is feasible, safe, and occasionally performed in patients with ICMP, (II) no adverse events associated with additional EVPP during LVAD implantation were found in acute or long-term follow-up, (III) once midventricular and coaxial alignment of the inflow cannula is achieved by implantation of the LVAD into the neoapex, pump position is maintained as suggested by our quarterly performed echocardiography, and (IV) prolongation of procedure and CPB time is acceptable in our presented cases and these combined procedures can be performed without cross-clamping in most patients.

Major advantages of a concomitant EVPP during LVAD implantation were already described^[12-14]^. Despite different reported techniques, prevention of thromboembolism by removal of thrombotic material from the former apex and more stable placement of the pump with coaxial and midventricular positioning of the inflow cannula towards the mitral valve are considered to be facilitated by EVPP. The herein described experience confirms these assumptions. Especially in preposterous dilated aneurysms (like the herein described 98 mm aneurysm with impending rupture) of the left ventricular apex, placement of an LVAD pump is not feasible. With the described technique including excision of thin myocardial wall, identification of vital myocardium, and removal of thrombotic material, shaping of an adequate neoapex for stable pump placement was feasible in all patients. Durability of this solution is of special importance in this scenario and preserved cannula position was confirmed in all patients during echocardiographic follow-up examinations. However, larger patient cohorts are needed before general recommendations regarding EVPP techniques can be made. Also, removal of thrombotic material was effective in the herein described patient cohort. Despite omission of cross-clamping in 80% of the patients, no strokes or pump failure occurred during follow-up. One patient presented with recurrent episodes of pump thromboses, which were treated by lysis therapy and are most likely not connected to preexisting thrombotic material in the left ventricle, since the first pump thrombosis occurred on postoperative day 560. The patient is now event free for 370 days. In the herein reported follow-up, so far the longest for these combined procedures, no events were seen connected to EVPP, which is further corroborating the safety and feasibility of the LVAD and EVPP approach in the instance of left ventricular apical aneurysm. Documented events were mainly bleeding and renal and respiratory failures. These complications were described extensively for patients with end-stage HF undergoing LVAD implantation^[15,16]^ and are not attributable to the performed Dor plasty.

## CONCLUSION

In conclusion, LVAD implantation with concomitant EVPP procedural is safe and feasible in patients with end-stage HF, ICMP, and apical aneurysms, facilitates pump placement by building a stable neoapex, and does not affect outcomes in terms of occurrence of acute and long-term events. These findings have to be confirmed in larger patient cohorts before general recommendations can be made.

**Table:** 

Author's roles & responsibilities	
AS	Substantial contributions to the conception or design of the work; or the acquisition, analysis, or interpretation of data for the work; drafting the work or revising it critically for important intellectual content; final approval of the version to be published
YS	Substantial contributions to the conception or design of the work; or the acquisition, analysis, or interpretation of data for the work; drafting the work or revising it critically for important intellectual content; final approval of the version to be published
LC	Substantial contributions to the conception or design of the work; or the acquisition, analysis, or interpretation of data for the work; drafting the work or revising it critically for important intellectual content; final approval of the version to be published
BS	Substantial contributions to the conception or design of the work; or the acquisition, analysis, or interpretation of data for the work; drafting the work or revising it critically for important intellectual content; final approval of the version to be published
YA	Substantial contributions to the conception or design of the work; or the acquisition, analysis, or interpretation of data for the work; drafting the work or revising it critically for important intellectual content; final approval of the version to be published
MR	Substantial contributions to the conception or design of the work; or the acquisition, analysis, or interpretation of data for the work; drafting the work or revising it critically for important intellectual content; final approval of the version to be published
MJB	Substantial contributions to the conception or design of the work; or the acquisition, analysis, or interpretation of data for the work; drafting the work or revising it critically for important intellectual content; final approval of the version to be published
HG	Substantial contributions to the conception or design of the work; or the acquisition, analysis, or interpretation of data for the work; drafting the work or revising it critically for important intellectual content; final approval of the version to be published
HR	Substantial contributions to the conception or design of the work; or the acquisition, analysis, or interpretation of data for the work; drafting the work or revising it critically for important intellectual content; final approval of the version to be published
SAP	Substantial contributions to the conception or design of the work; or the acquisition, analysis, or interpretation of data for the work; drafting the work or revising it critically for important intellectual content; final approval of the version to be published
AMB	Substantial contributions to the conception or design of the work; or the acquisition, analysis, or interpretation of data for the work; drafting the work or revising it critically for important intellectual content; final approval of the version to be published
